# The role of integration and clonal expansion in HIV infection: live long and prosper

**DOI:** 10.1186/s12977-018-0448-8

**Published:** 2018-10-23

**Authors:** Elizabeth M. Anderson, Frank Maldarelli

**Affiliations:** HIV Dynamics and Replication Program, NCI, NIH, Frederick, MD 21702 USA

**Keywords:** HIV persistence, HIV reservoirs, Proviral integration, Clonal expansion

## Abstract

Integration of viral DNA into the host genome is a central event in the replication cycle and the pathogenesis of retroviruses, including HIV. Although most cells infected with HIV are rapidly eliminated in vivo, HIV also infects long-lived cells that persist during combination antiretroviral therapy (cART). Cells with replication competent HIV proviruses form a reservoir that persists despite cART and such reservoirs are at the center of efforts to eradicate or control infection without cART. The mechanisms of persistence of these chronically infected long-lived cells is uncertain, but recent research has demonstrated that the presence of the HIV provirus has enduring effects on infected cells. Cells with integrated proviruses may persist for many years, undergo clonal expansion, and produce replication competent HIV. Even proviruses with defective genomes can produce HIV RNA and may contribute to ongoing HIV pathogenesis. New analyses of HIV infected cells suggest that over time on cART, there is a shift in the composition of the population of HIV infected cells, with the infected cells that persist over prolonged periods having proviruses integrated in genes associated with regulation of cell growth. In several cases, strong evidence indicates the presence of the provirus in specific genes may determine persistence, proliferation, or both. These data have raised the intriguing possibility that after cART is introduced, a selection process enriches for cells with proviruses integrated in genes associated with cell growth regulation. The dynamic nature of populations of cells infected with HIV during cART is not well understood, but is likely to have a profound influence on the composition of the HIV reservoir with critical consequences for HIV eradication and control strategies. As such, integration studies will shed light on understanding viral persistence and inform eradication and control strategies. Here we review the process of HIV integration, the role that integration plays in persistence, clonal expansion of the HIV reservoir, and highlight current challenges and outstanding questions for future research.

## Background

Despite the success of combination antiretroviral therapy (cART) to block viral replication and halt disease progression, HIV viremia persists in the blood and anatomic compartments for years after therapy is initiated [1]. Although current therapies improve morbidity, mortality, and quality of life [2–5], long-term cART is associated with drug toxicities and persistent immune activation that contributes to morbidity and mortality, including a higher risk for non-AIDS related diseases including cardiovascular disease, cancer, kidney disease, liver disease, neurologic disease, and bone diseases [3, 6, 7]. Furthermore, if antiretroviral treatment is interrupted, viremia rebounds to near pre-therapy levels within weeks in most patients [8–10]. As a consequence, developing strategies to eradicate or control HIV without antiretroviral therapy are a high priority [11]. HIV rebounds from a reservoir of latently infected cells and consistent with this, the rebounding virus is archival in nature [12]. The source of persistent residual viremia that gives rise to rebounding virus upon treatment interruption remains largely unknown and is paramount for HIV cure initiatives.

A hallmark of retroviruses, and a key step in the HIV replication cycle that enables viral persistence, is the integration of the HIV DNA into the host genome. Integration is a multistep process that involves both viral and host factors resulting in a stable and irreversible positioning of the double stranded reverse transcription product, the provirus, within the host cell. Integration does not require that the viral DNA be replication competent or even full length, and integration may proceed with highly deleted genomes. The choice of location of the retrovirus integration site within the host genome is neither entirely random nor specifically targeted. Integration preferences for various retroviruses have been identified and influence locations within the host genome where proviral integration takes place [13, 14]. Upon integration, the HIV provirus persists for the life of the cell and transcription of viral mRNA is coordinated by host cellular mechanisms. HIV primarily infects activated CD4+ T cells, a small subset of which may transition back to a resting memory state that is non-permissive for viral gene expression [15]. Although resting cells largely restrict productive HIV infection (reviewed by Zack et al. [16]), HIV can directly infect resting cells in vitro [17, 18] providing an alternative mechanism for establishing latency. In either case, a reservoir of latently infected cells may be unaffected by host immune responses and have a very long half-life [19–22].

HIV integration into long-lived cells represents an intrinsic characteristic that is central to HIV persistence and therefore a major barrier to an HIV cure or control strategy. During cART, lymphocyte populations undergo substantial change as ongoing HIV transmission is blocked, and a degree of immune restoration occurs. The population of HIV infected cells is molded over time since these cells may persist, be lost, or undergo clonal expansion. Understanding immune and viral mechanisms responsible for persistence is essential to characterizing the population of infected cells harboring replication-competent HIV that remain on therapy for prolonged periods and are a primary objective of control and eradication.

The only HIV reservoir that gives rise to rebounding virus, making a cure unachievable as of yet, is the reservoir of replication competent proviruses. Although over 95% of all integrated proviruses are defective or deleted, a small fraction of inducible replication competent proviruses persist for years on cART [23]. Still, defective and deleted proviruses are capable of producing viral proteins which can be targeted by the immune system and may contribute to persistent immune activation and long-term HIV pathogenesis [24, 25]. The majority of replication competent HIV proviruses persist in resting CD4+ T cells of a memory phenotype [21]. Since HIV gene expression is dependent on host transcription factors that are present only during cellular activation, HIV transcription is nearly silenced in resting CD4+ T cells. This results in a stably integrated yet transcriptionally silent provirus that will persist for the life of the cell, and can be reactivated to produce infectious virus. Resting CD4+ memory T cells have a very long half-life [19] and even after years on cART, resting CD4+ memory T cells can maintain themselves in a quiescent state or through periodic cell division without reactivation of the latent virus.

The HIV reservoir is established early during primary infection and is remarkably stable with a half-life of 43–44 months [26, 27]. As a consequence, current suppressive therapies must be maintained in an individual for over 70 years to achieve complete elimination of the reservoir. Similarly, HIV DNA levels remain detectable and are stable in most patients after years on suppressive therapy [28]. HIV reservoir half-life determinations vary substantially, in part due to technical approaches. Measurements of HIV DNA vary according to the HIV proviral target measured, for example LTR compared to *gag*. Determining the number of cells with infectious HIV proviruses may vary depending on the distinct quantitative viral outgrowth assay in use [29]. Understanding the underlying mechanisms that determine the variability in reservoir half-life will shed light on how the reservoir decays and whether immune selection pressure influences the rate of decay. The intrinsic stability of the reservoir indicates that its long term maintenance is a major mechanism that supports HIV persistence. The latent reservoir can be maintained over the course of cART through periodic homeostatic proliferation and through clonal expansion of HIV infected cells, both antigen mediated and integration site driven (reviewed by Murray et al. [30]). Additionally, promotion of cell survival through antiapoptotic regulation (reviewed by Badley et al. [31]) or the integration of proviruses into certain genes may also enable cells harboring integrated proviruses to persist for prolonged periods. Targeting the mechanisms for reservoir maintenance may provide novel curative strategies to deplete the latent reservoir.

Fundamental to bridging knowledge gaps towards HIV eradication is an understanding of the establishment and maintenance of cellular reservoirs and their persistence. The dramatic example of HIV cure [32, 33], as well as accumulating reports of post treatment control without cART [34–38] suggests that viral eradication or long-term viral remission may be achievable. Further study of proviral integration and persistence will aid in the development of novel strategies towards an HIV cure. A number of reviews on integration details have been published in the last several years that summarize aspects of integration and persistence including integrase structure and enzymology [39, 40], recent methods of detecting and quantitating integration sites [41, 42], as well as studies on other retroviruses integration that have useful insights for understanding persistence of HIV infected cells [43]. Here, we review concepts and controversies regarding HIV integration and clonal expansion of infected cells in the setting of current understanding of host cell populations, and highlight unanswered questions for future research.

## Dynamics of HIV infected populations

### Establishing a reservoir for HIV

Characterizing HIV persistence during prolonged cART requires a fundamental understanding of infected cell populations and their dynamics in infected individuals during cART. HIV infects numerous host cell types in diverse anatomic compartments typical of cells of lymphocyte [44] and myeloid lineage [45]. Various CD4+ T cell subsets are infected, but only some are likely sources of long-term persistence. Activated cells are typically infected by HIV and frequently undergo cell death from viral induced or immune elimination. Infrequently, activated CD4+ T cells infected with HIV transition to a resting memory state that is only poorly permissive for viral gene expression if at all [20, 21, 46]. These latently infected cells have a very long half-life [19] and in the absence of any viral gene expression may evade host immune responses. New studies to address whether cells remain latent permanently and whether they may evade immune surveillance are necessary. The memory T cell pool is composed of two main compartments, central memory (T_CM_) and effector memory (T_EM_) T cells, which are characterized by their homing abilities and effector functions [47, 48]. An intermediate compartment has also been described and is designated as the transitional memory compartment (T_TM_). Both T_CM_ and T_EM_ compartments persists for decades [49] however the kinetic behaviors of these populations differ [50]. T_CM_ have a high proliferative capacity and are long-lived [51]. On the other hand, T_EM_ are rapidly turned over constituting a short-lived population with an extremely low proliferative capacity [50].

Another potential contributor to the HIV reservoir is a less differentiated subset of long-lived memory T cells with a high self-renewal capacity known as stem-cell memory CD4+ T cells (T_SCM_) [52]. T_SCM_ can be differentiated from naïve T cells via TCR stimulation in vitro supporting the idea that naïve T cells represent the precursor to T_SCM_. T_SCM_ retain many phenotypic characteristics of naïve T cells (CD45RA+ and CCR7+) but additionally express memory T cell markers including CD95 and CD62L [52]. T_SCM_ are infected by HIV in vitro, however, only a small fraction of cells are able to support productive infection [53]. Still, prolonged survival of T_SCM_ indicate that they may become the dominating population in the reservoir after long term suppression when ongoing rounds of virus replication are halted and other memory T cell compartments decay. Indeed, Buzon et al. found T_SCM_ infected cells contribute minimally after 1 year on suppressive therapy but their contribution increased after long term therapy [54]. These findings suggest that HIV infected T_SCM_ cells could comprise a viral niche that promotes long-term viral persistence. Furthermore, replication competent virus has been recovered from CD45+/CD62L+ memory T cells ex vivo [55]. Given the potential for T_SCM_ to survive for prolonged periods and maintain a high proliferative capacity, it is critical to determine the contribution of the T_SCM_ compartment to the HIV reservoir.

Recent reports suggest additional helper T cell populations are infectable by HIV. As described by Lichterfeld and coworkers, these additional T cell populations express sufficient CXCR4 (Th1, Th17), or CCR5 (Th2 and Th9) to be infected in vitro by X4 and R5 tropic HIV respectively [56]. Extensive cell sorting studies recovered HIV DNA from these subsets in HIV infected individuals, indicating they are infected in vivo. The longevity of these subsets remains uncertain, but they are reported to have long half-lives, and thus may represent relevant reservoirs for HIV infection.

Other cell lineages, including tissue resident cells may be infected with HIV and may represent important sources of persistence of HIV infected cells during cART [57]. Intriguingly, HIV infection occurring at the stem cell level [58–60] raises the possibility that other downstream lymphocyte lineages, including B cells, may contain HIV proviruses. Although routine analyses of B cells does not typically detect HIV proviruses, infection may be present at levels below assay limits. Collins et al. have reported the presence of such cells as well as hematopoietic stem cells (HPC) infected with HIV at low frequency [61] and have suggested HPC infection may occur in only a subset of patients [60]. Others have published conflicting reports on the presence of HIV infected HPC in vivo [62, 63]. It is essential to determine if long-lived hematopoietic stem cells also contribute to the HIV reservoir. Additional potential reservoirs for HIV infection have been reported in myeloid lineages, including brain macrophages [64–66] and astrocytes [67, 68] in the central nervous system, and podocytes in the kidney [69]. The relevance of HIV infection of these long-lived cells as reservoirs for HIV infection during long-term cART is actively under investigation.

### Maintaining a reservoir of HIV infected cells during cART

Longstanding untreated infection is characterized by progressive loss of lymphocytes with a preferential decline in CD4+ cells, and consequently a decrease of CD4/CD8 ratios. As described above, not all HIV infected cells are rapidly eliminated. The proportion of all lymphocytes that are infected is relatively low (1:100–1:1000). As such, the progressive loss of CD4 cells that is characteristic of untreated HIV infection is not due to direct viral killing per se, but to associated mechanisms, such as bystander effects and activation-induced cell elimination. Long-lived infected cells are less frequent, and are revealed upon initiating cART. The frequency of HIV DNA+ cells declines within the 1–4 years on cART, but remains relatively stable thereafter within the range of 1–3 per 10,000 CD4 cells during therapy [28]. As reviewed in this Special Issue by Pinzone and O’Doherty [70], determining levels of integrated HIV DNA can shed light on how reservoirs are maintained during cART. Prior to treatment initiation, total and integrated HIV DNA levels are higher in individuals treated during chronic HIV infection and decrease to a lower extent than those treated in primary HIV infection [71–73]. Furthermore, integrated HIV DNA continues to decay after prolonged therapy in individuals treated during primary infection suggesting that enhanced immune responses in these individuals are able to clear HIV infected cells more effectively [72, 74, 75]. During this same period, CD4 cell numbers typically increase with a measure of immune restoration. Thus, the number of infected cells keep pace with the overall recovery of CD4 cells. During prolonged cART, infected cells persist, are lost, or undergo clonal expansion in the context of a dynamic (and aging) immune cell population. It is not known how the proportion of infected cells remains stable as CD4 numbers rise, but it is likely that infected cells respond to immune signals to persist and proliferate. As such, the abundance of HIV infected cells in T cell subsets during cART may be continually molded by immune forces. The factors driving the maintenance of infected cells is of critical interest in understanding persistence and have been broadly divided into homeostatic and direct immune stimulatory factors [76].

Latently HIV infected resting memory CD4+ T cells can undergo homeostatic proliferation and antigen mediated or integration site driven clonal expansion [77–80] which may maintain the reservoir during cART. T cell homeostasis is a state of equilibrium maintained through self-regulation of T cell pools. T cells present in circulation and residing in tissues provide afferent and efferent immune arms that are central to both adaptive and innate immune responses. T cell homeostasis is mediated by homeostatic cytokines that belong to the common γ chain cytokine family including IL-2, IL-4, IL-7, IL-9, IL-15, and IL-21. A strong inverse correlation between baseline CD4 count and IL-7 plasma levels has been described, but the factors associated with this correlation have not been identified [81–83]. Lymphocyte population dynamics has been directly investigated using in vivo bromodeoxyuridine (BrdU) labeling. These studies found that the increase in IL-7 is the result of CD4 depletion, but is not the primary driver of CD4 proliferation in the context of HIV infection [84]. Conversely, IL-15 controls survival and turnover of memory CD4+ T cells. Patients with advanced HIV infection have increased type I IFN plasma levels. Ongoing exposure to homeostatic forces and type I IFN activation may be responsible for selective depletion of CD4+ T cells [85]. IL-7 increases the number of CD4+ T cells by promoting their survival and proliferation, providing a rationale for IL-7 treatment to assist immune reconstitution in the setting of HIV infection [86, 87]. However, IL-7 induces proliferation without virus reactivation indicating that homeostatic proliferation can maintain the reservoir over time [88, 89].

Antigenic stimulation driven either by specific common antigens (CMV, EBV, HPV) or nonspecific immune activators, such as bacterial cell products translocated across the leaky gut wall that is present in HIV infection, may induce generalized immune activation and could ultimately contribute to the clonal expansion of HIV infected cells. IL-2 is produced by CD4+ T cells following activation by an antigen and drives T cell proliferation [90]. It is possible that HIV infected cells can undergo clonal expansion in response to cognate or cross reacting antigens. HIV-specific CD4+ T cells are a favored target for HIV infection [91]; it is likely these HIV specific cells persist during therapy, and that low level HIV production during cART may continue to drive persistence and expansion of these specific subsets. Other antigens commonly encountered (e.g., CMV, EBV) may also represent potential sources of clonal expansion. We previously reported a cell clone that was widely anatomically distributed, but significantly enriched in cancer metastases, suggesting that these cells proliferated in response to the cancer antigen [79]. Specific T cell receptor analyses were not possible in this single example. Advances in T cell receptor characterization of individual HIV infected cell clones will be critical for understanding the role of antigen driven clonal expansion on shaping the proviral landscape. These different mechanisms can promote cellular clonal expansion to maintain or potentially increase the size of the latent reservoir of intact replication competent proviruses.

HIV infection is characterized by a state of chronic immune activation which may play a strong role in maintaining persistence and clonal expansion of HIV infected cells. Prior to cART, viremia is substantial and activated CD4+ T cells infected with HIV die rapidly with a half-life of approximately 1.5 days which can be attributed to a variety of cytopathic effects. During chronic HIV infection and in the absence of treatment, abortive infection leads to the release of inflammatory cytokines that contribute to chronic inflammation, CD4+ T cell depletion, dysregulation of T cell homeostasis and ultimately AIDS [92, 93]. Even after the introduction of cART, low level viremia persists likely as the result of the stochastic reactivation of latently infected cells [94], infected cells are slowly eliminated [26, 27], but HIV antigens continue to persist thereby potentially contributing to chronic immune activation and dysregulation [95, 96]. Previous work measuring the decay kinetics of integrated HIV DNA from individuals treated during chronic HIV infection suggest diminished immune responses could promote persistence with the inability to effectively eliminate HIV infected cells during therapy. We recently found HIV infected cells harboring proviruses that contain internal HIV genes (such as *gag*) decline at a faster rate than *gag*-lacking proviruses upon cART initiation [76]. These findings further suggest a potential role for immune pressure to shape the proviral landscape during cART. Finally, in addition to generalized systemic immune activation, HIV mediated inflammation may be anatomically restricted [97]. Understanding the forces driving persistence and clonal expansion of resident T cells in tissues will shed important light on the mechanisms of HIV persistence and pathogenesis in vivo.

### Detecting reservoirs of HIV infected cells and their turnover

Recent lines of research may improve our understanding of lymphocyte kinetics, and critical advances for quantifying HIV reservoirs are essential (Reviewed in this Special Issue by Wang et al. [98]). The simplest way to determine the viral burden in various cell subsets uses standard PCR-based techniques that measure total HIV DNA but is unable to distinguish integrated from unintegrated forms of HIV DNA. The utility of measuring integrated HIV DNA to understand how reservoirs are formed and persist is reviewed in this Special Issue by Pinzone and O’Doherty [70]. To date, HIV DNA has been measured in total peripheral blood mononuclear cells (PBMCs) [99], CD4+ T cells [100], resting CD4+ T cells [101], as well as in the gut-associated lymphoid tissue (GALT) [102, 103]. Recent approaches have used a next generation platform of PCR called droplet digital PCR (ddPCR) (Reviewed in this Special Issue by Rutsaert et al. [104]). ddPCR utilizes absolute quantification rather than relative quantification based off extrapolating from a standard curve in traditional qPCR. Eliminating the error from user generated or instable standard curves enables ddPCR to be more accurate than qPCR [105]. Furthermore, PCR inhibition is limited since the bulk PCR reaction is partitioned into circa 20,000 individual reactions. ddPCR has been used to quantify total HIV DNA in vivo from PBMCs, CD4+ T cells, T regulatory (Treg) cells, and in cells from cerebrospinal fluid [29, 106–110].

Despite these advances, total HIV DNA quantification using standard PCR-based techniques has been shown to be at least two orders of magnitude higher than latent reservoir size measurements using the quantitative viral outgrowth assay (qVOA), the gold standard technique to measure the replication competent reservoir [29]. This large discrepancy is likely due to the fact that the majority of integrated proviruses are deleted [111], therefore total HIV DNA alone cannot provide an accurate estimate of latent reservoir size. Still, HIV DNA levels remain an important biomarker for viral persistence [112] and can predict viral rebound upon treatment interruption [9, 113]. Moreover, HIV DNA levels strongly correlate with qVOA thereby providing a surrogate marker for the size of the latent reservoir using an inexpensive and less time consuming approach [29, 114]. New duplexed ddPCR strategies that quantify internal targets may improve the accuracy of amplification methods to quantify replication competent reservoirs [115].

Understanding lymphocyte dynamics and turnover is a second critical area requiring advancement. In the context of HIV infection, persistent immune activation is associated with an increase in cell proliferation and cell death. In vivo labeling can provide reliable measurements of cell turnover and proliferation. Labeling newly synthesized DNA with deuterium provides a method for directly measuring turnover in a population of cells, with the caveat that minority populations cannot be studied easily. BrdU is a thymidine analogue that is incorporated into the DNA of replicating cells and can subsequently be detected by flow cytometry with a monoclonal antibody [116]. In vivo BrdU labeling identified two populations of CD4 and CD8 T lymphocytes which can be characterized as either rapidly proliferating or slowly proliferating [117]. Activated cells have the highest proliferative rates, followed by effector and central memory, and naïve cells have the lowest proliferative rates [84]. Increased CD4+ T cell turnover is associated with higher HIV plasma RNA levels and increased CD4 depletion, suggesting that lymphocyte turnover is a direct consequence of HIV infection [117]. Additionally, immune responses also play a role in the turnover of most CD4 and CD8 memory cell subsets [84]. On the other hand, turnover of the naïve compartment can be attributed to homeostatic mechanisms rather than immune mediated activation [84]. Long term labeling with deuterated water found T cell subpopulations possess distinct half-life characteristics and that T cells died more rapidly in individuals with advanced HIV infection [118]. Continuing research to measure the turnover of cells, including HIV infected cells, in these subsets is crucial to determine the longevity of these compartments and their role in promoting long-term persistence of HIV infected cells.

Further definition of the spectrum of cell subsets infected by HIV is also essential. Novel single cell and transcriptomic studies [119–122], as well as quantitative studies of populations of CD4 and CD8 cell subsets are advancing our understanding of human immune response to pathogens, including chronic infections, and may potentially inform the status of HIV infected cells with integrated proviruses. To date, single cell methods have been useful in characterizing the fate of T cells [122]. Understanding the functionality and dynamics of T cell populations over prolonged periods as individuals age is especially germane [123–125]. Since the frequency of HIV infected cells during cART is low, functional studies of T cells infected with HIV necessitates innovative approaches that overcome technical challenges to characterizing individual infected cells.

## Integration: the central event in HIV replication

The integration of the HIV provirus into the host genome is a key characteristic of retroviruses and an essential step in the HIV life cycle that enables viral persistence. Prior to integration, the virally encoded enzyme reverse transcriptase (RT) synthesizes a linear double-stranded cDNA intermediate from the viral RNA genome. This reverse transcription product is the substrate for integration and contains homologous long terminal repeat (LTR) sequences at both the 5′ and 3′ ends [126]. The process of integration is the product of a viral enzyme, integrase, but interactions with other viral and cellular factors are required for successful integration to take place in an in vivo setting.

### Integrase structural and enzymatic studies

**Fig. 1:**
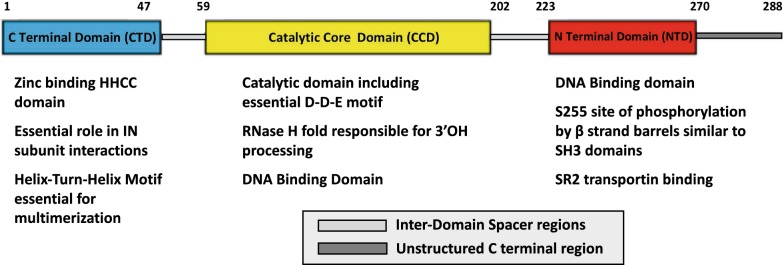
Structural domains and function of HIV integrase

Integrase (IN) is a member of the transposase family of nucleotidyl transferases (E.C. 2.7.7) that catalyze the transfer of 3′ OH ends of HIV DNA to a host DNA acceptor. IN has a tripartite structure consisting of an N terminal Domain (NTD), a catalytic core domain (CCD) and a C-terminal domain (CTD). NTD and CTD have important functions coordinating interactions with DNA and chromatin binding. CCD contains enzymatic activity, including a D, D, E active site motif that is found in a number of nucleotidyl transferases, which coordinates essential divalent metal cations necessary for catalysis (Fig. 1).

**Fig. 2:**
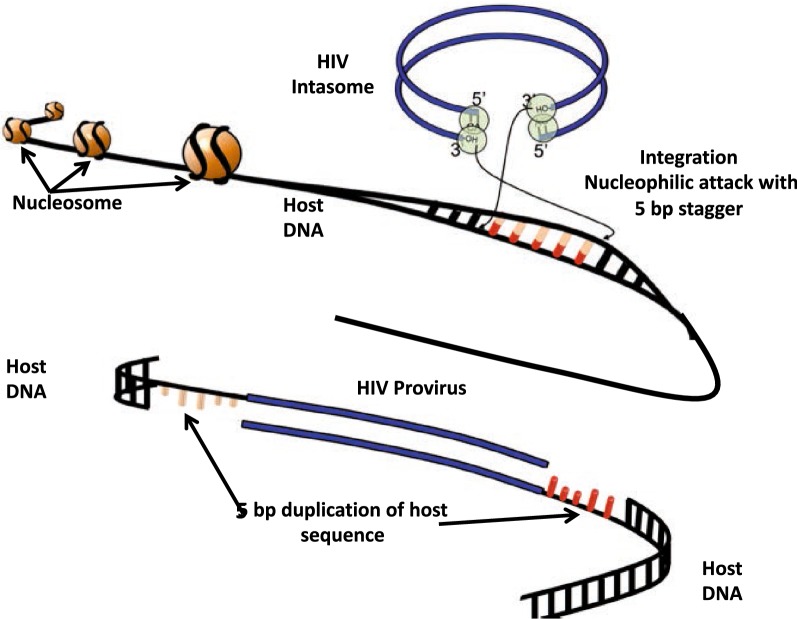
Depiction of the integration of HIV proviral DNA into the host genome

Understanding the structure and function of integrase has been critical to explain the establishment of the provirus and for developing integrase inhibitors. The structure of HIV integrase has been the subject of intense investigation; crystals of the catalytic portion of HIV IN have been available for years [127], but the full length enzyme has had technical issues [128, 129]. Fortunately, pivotal studies of foamy virus and maedi-visna virus integrase have greatly advanced the field [130–132] and revealed critical structural characteristics of integration [133, 134]. These studies utilized crystallographic approaches of integrase and DNA substrate co-crystals and cryo-electron microscopy (cryo-EM) approaches of integrase multimers and DNA. Structural studies combined with biochemical studies using in vitro assays of purified HIV IN enzyme and host DNA have characterized the multistep process of HIV integration (Fig. 2). HIV IN multimers are positioned at the ends of DNA product. The initial structure, denoted the intasome or stable synaptic complex, is poised to initiate the multistep integration reaction, beginning with an IN-mediated 2 nucleotide deletion at the 3′ end of each viral DNA molecule, creating staggered ends on the viral substrate for subsequent integration into the host DNA.

The stoichiometry of Integrase:DNA has been a subject of intense interest to discern the processes that coordinate the integration reaction. Furthermore, specific inhibitors that disrupt multimerization are currently in therapeutic development. A number of studies have suggested that IN from HIV and other retroviruses assumes a quaternary structure at the ends of the proviral DNA molecules [133]. Over the last several years, the development of cryo-EM has revolutionized the visualization of large macromolecular assemblies. Cryo-EM has permitted the visualization of HIV IN structure that has not been previously possible through traditional crystallographic approaches. Intriguing new cryo-EM studies have identified structures for HIV IN containing more than four IN molecules. The relative contributions of these higher order structures to integration and interactions with elements of the PIC remain uncertain and are topics of active investigation [135]. The development of a new class of IN inhibitors, called allosteric integrase inhibitors (ALLINIs), will be particularly useful probes in understanding the role of higher order structures in HIV IN (reviewed by Feng et al. [136]). ALLINIs bind at the IN dimer interface resulting in aberrant IN multimerization, with a number of critical consequences for HIV replication, including the production of aberrant particles with viral ribonucleoprotein eccentrically localized in virions [136]. These defective virions have reduced reverse transcriptase activity and accelerated decay rates of viral RNA in subsequent rounds of replication [137–140]. Thus, disruption of proper IN multimerization has consequences for both early and late steps in HIV replication.

The details of binding and cutting host sequences has been extensively studied in model systems. In cryo-EM studies of maedi-visna integrase, tetramers assembled at each DNA end (with 2 nucleotides at the 3′ end of each viral DNA molecule already removed), then the CTDs bind in expanded major grooves of DNA targets effectively bending the target DNA [141]. Once bound, a target capture complex cuts the host DNA with a 5 nucleotide staggered cut yielding the strand transfer complex (Fig. 2) (for details see Lesbats et al. [142]), enabling transfer of the viral DNA to host cell DNA. The intervening 5 nucleotide gap is filled in by host DNA polymerase, and ligated by host ligase. One consequence of the 5 nucleotide staggered cutting mechanism of the host DNA by integrase is the duplication of these 5 nucleotides of host sequence directly flanking the 5′ and 3′ ends of the provirus, which provides a useful assay to confirm authentic integrations when both the 5′ and 3′ sites have been sequenced.

The extraordinary detail afforded by crystallographic and cryo-EM studies combined with an extensive understanding of IN enzymology, the role of specific domains involved in IN enzymatic activity (Fig. 1), and the effects of type I and II mutations provides a strong foundation for understanding the role of IN in HIV replication and identifying new avenues for HIV IN therapeutic development.

### Determinants of integration site selection

In in vitro assays of purified integrase, integrases show little host site specificity, with the exception of weak palindromic sequences at target sites [14]. In contrast, analyses of integration site distribution in retroviral tissue culture infections and in samples from animal studies or patients reveal integration site preferences that highly influence the overall infection program. Preferences are exercised by the cellular partners that the intasome engages during the transport to the nucleus and integration. As described by Ciuffi [143], Craigie [144], and Debeyser [145, 146], these factors may be categorized as those with chaperone-like activity and those with chromatin-tethering activity. Understanding nuclear import and chromatin association is essential to understanding distribution of integration sites, as interactions with tethering and chaperone partners may have direct and indirect effects on distribution of integration sites. Cofactors for integration have been investigated for a number of retroviruses. As reviewed by Engleman [147], some retroviruses have distinct integration preferences, while others remain relatively random. Here we will review data for HIV.

Unlike many retroviruses, HIV infects non-dividing cells, requiring import of the reverse transcript into the nucleus which takes place in the context of a large multimeric pre-integration complex (PIC). PIC contents remain under study (reviewed in Suzuki & Craigie [148] and Craigie & Bushman [144]), as understanding the composition of the PIC will provide insights into requirements for initial steps in establishing the proviral state and potential targets for interruption in non-dividing cells. Viral components include HIV RT, IN, and an uncertain portion of the complement of HIV CA from the incoming core are associated with the PIC. Cellular proteins interacting with the PIC include the barrier to autointegration factor 1 (BAF1), high mobility group proteins (HMG), lamina-associated polypeptide 2α (LAP2α), lens-epithelium-derived growth factor (LEDGF/p75), and the karyopherin transportin SR2 (TRN-SR2, TNPO3). TNPO3 binds directly to the CCD and CTD of IN [149] and may participate in shuttling the PIC to the nucleus. The size of the PIC is uncertain, but it must fit through the nuclear pore, and the process of import is essential yet remains unclear. As IN associates with the ends of the HIV DNA, the internal HIV sequence need not be full length, and can be defective or deleted, making the HIV proviral makeup in an individual highly diverse. As reverse transcription may take place in the nucleus [150], RT and associated factors may clearly be imported into the nucleus.

A number of critical outstanding questions defining the early events of infection are currently under study. These include the requirements for uncoating and transport, the composition of PIC structures, the factors required for intracellular transport and nuclear import, the coordinated involvement of cellular and nuclear cytoskeletal structures, as well as the overall kinetics and rate limiting steps of the process. A number of factors have been reported to be involved in regulating import, including nuclear membrane proteins SUN1 and SUN2 [151]. The central role for integration in HIV replication makes it an attractive target for therapy. Enzymatic inhibitors have been highly successful, allosteric inhibitors or agents that interrupt other integrase functions such as multimerization or interactions with cellular proteins have already yielded interesting candidates for further study [136, 152–154]. Critical advances in tracking single particles with elegant microscopic approaches have begun to characterize the kinetics of nuclear import [150, 155].

Once nuclear import has been accomplished the provirus can integrate into the host genome. The site of proviral integration for retroviruses is relatively nonspecific, with general preferences among the orthoretrovirinae subfamily. For HIV, integration site preferences include actively transcribed genes, gene rich regions of chromosomes, introns over exons, and generally exclude promoter regions. As introns are typically much larger than exons, the excess integrations into introns is likely due to larger overall size of introns rather than a functional constraint or preference per se. Preferences for activated genes [156] are generally mediated by cellular cofactors that bind IN [157]. As described by Ciuffi [158] and Debeyser [145, 146], these factors may be categorized as those with chaperone-like activity that are primarily involved in nuclear import, and those with chromatin-tethering activity.

Chief among the factors coordinating binding to chromatin is the transcriptional activator LEDGF/p75 [159]. Co-crystal studies identified contacts between the integrase CCD and CTD of two IN molecules and the C-terminal integrase binding domain (IBD) in LEDGF/p75 [132]. These findings suggest that LEDGF/p75 forms a bridge between the NTD domain of one IN dimer and two CCD domains of a second dimer [132, 143, 160]. The LEDGF/p75 N-terminal domain contains an AT-hook motif which mediates DNA-binding at AT-rich regions [143], and a PWWP domain that mediates binding to chromatin [161]. LEDGF/p75 knock down experiments showed no decrease in the ability of HIV DNA to integrate into the host genome, but revealed shifts the integration site distribution away from transcriptionally active and AT-rich regions [159]. In a series of domain swapping experiments, Hughes and coworkers demonstrated that replacing the AT hook and PWWP domains of LEDGF/p75 with the chromatin binding domains of proteins having euchromatin or heterochromatin binding specificities redirects integration according to the specificity of the heterologous binding domain [162]. These studies highlight the critical role of LEDGF/p75 and demonstrate approaches to manipulate integration that may be useful in the design of safer retroviral vectors [162].

Recent reports have investigated the role of nuclear architecture in integration preferences. HIV enters via nuclear pore complexes (NPCs) into regions that are typically euchromatin rich as a result of Tpr, a protein constituent of the NPC basket region that facilitates heterochromatin exclusion zones [163]. Tpr knock down results in chromatin reorganization and no exclusion of heterochromatin from NPC regions, but does not reduce HIV integration although HIV transcription is significantly impaired [159, 164]. These findings indicate that in the absence of Tpr, HIV integration continues directly after or in concert with nuclear import but into regions that are unfavorable for HIV transcription [164]. Marini et al. analyzed the topologic distribution of HIV integration sites and reported highest levels of integration in genes located near NPCs with a decreasing gradient of integration in genes at greater distance from the nuclear envelope [165]. There are a number of techniques to localize HIV proviruses within nuclei: labeling of nascent HIV DNA with 5-ethynyl-2′-deoxyuridine (EdU) and immunofluorescent detection [166], identifying integrated proviruses by immunolocalization of endonucleases that introduce specific double strand breaks in HIV [167], detecting HIV proviruses in live cells using quantum dot labeled Transcription Activator-Like Effectors (TALEs) [168], colocalizing HIV Tat with HIV LTRs of integrated proviruses in isolated live nuclei [169], and detecting HIV IN live cells using specific immunofluorescent [170]. These studies have identified HIV proviruses or HIV IN near the nuclear membrane after import. Other studies reported HIV signal at some distance from nuclear membrane [150, 166, 168], while real-time studies from Burdick et al. demonstrated slow movement away from NPCs [170].

Hope and coworkers have suggested studies to investigate the role of nuclear architecture, other HIV proteins (e.g., capsid), and cellular components in HIV integration [171]. Such studies may reveal useful insights into HIV replication and nuclear import, especially regarding how the processes of reverse transcription and nuclear import are coordinated. These approaches will require analysis of the primary targets of HIV, including lymphocytes and macrophages. While macrophages have comparatively large nuclei and are likely easier to analyze, new studies of lymphocytes are especially needed. Visualization approaches, including sensitive single cell technologies that can identify intranuclear location of HIV DNA within these nuclei are essential. Methods to simultaneously detect HIV provirus and HIV RNA transcription in infected lymphocytes have been reported [172]. Live cell studies are particularly useful to elucidate the dynamics of RNA expression from HIV proviruses [168–170].

Not all of the newly synthesized viral cDNA molecules, however, are successfully integrated into the host genome. In the nucleus, a subset of reverse transcripts comprise unintegrated episomal molecules that include 1- or 2-LTR circles and defective autointegrants [173]. Circular forms are not replicated as the cell divides, are diluted out upon cell replication, and do not contribute to ongoing replication. The longevity of such forms is a subject of debate. In tissue culture, circular LTR forms are lost several weeks after infection [174–176] but are stable in long term cultures of nondividing cells [176, 177]. In vivo they may persist for longer periods [28, 178], similar to T cell receptor excision DNA circles (TRECs) [179].

### Integration in vivo: analysis of HIV integration junction sequences

Initial in vivo studies of proviral integration sites utilized inverse PCR to characterize HIV integration sites in CD4+ T cells from HIV infected individuals [180–182]. These studies confirmed what had been found from in vitro tissue culture systems with a preference for HIV DNA to integrate into transcriptionally active genes, usually within introns (range: 93–96%) [180–182]. Initial longitudinal analyses revealed that identical integration sites could persist in individuals for years during therapy. However, the methods used could not determine whether this arose through clonal expansion or simply represented long-term persistence [181]. Multiple individuals were identified as having proviral integration sites in the *BACH2* gene and all integrations were in the same orientation of the gene [181]. *BACH2* is highly expressed in B lymphocytes and plays a role in the regulation of B cell development [183]. While expression of *BACH2* has been shown in T lymphocytes in vitro [183] and in vivo [181], the function of *BACH2* in these cells remains unknown. Further, it was not understood at the time if the enrichment of integration sites in *BACH2* is the result of preferential integration or, rather, a selective advantage towards long-term persistence of cells that harbor integrants in *BACH2*.

**Fig. 3:**
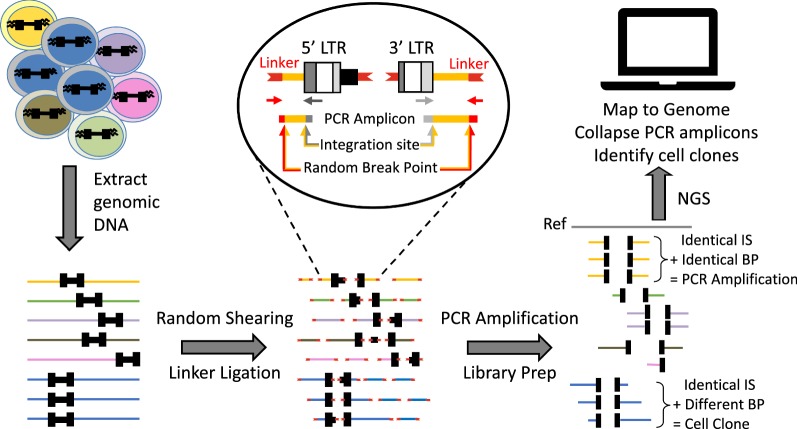
Linker mediated HIV integration site assay (ISA) workflow. Total genomic DNA is first extracted then randomly sheared by Covaris sonification into 300–500 bp fragments. Sheared fragments are end repaired and a single dA overhang is added, then linkers containing a single T overhang are ligated onto the sheared ends (red). The pop out displays the PCR amplification strategy to selectively amplify integration sites. Primers that are complementary to the 5′ HIV LTR in U3 (dark grey arrow) and the 3′ HIV LTR in U5 (light grey arrow) are combined with linker specific primers (red arrows). The resulting amplicons contain linker sequence, the random breakpoint (BP), and the HIV/host junction sequence at the integration site (IS). The amplicons are then subjected to Illumina Miseq paired end sequencing. Sequences obtained are run through a stringent bioinformatics pipeline to map the location of the integrated provirus against a reference host genome and to determine the distance to breakpoint. Identical integration sites from amplicons with different break points in the host genome are the result of clonally expanded cells, whereas identical integration sites from amplicons with identical break point distances arose during PCR amplification

**Fig. 4:**
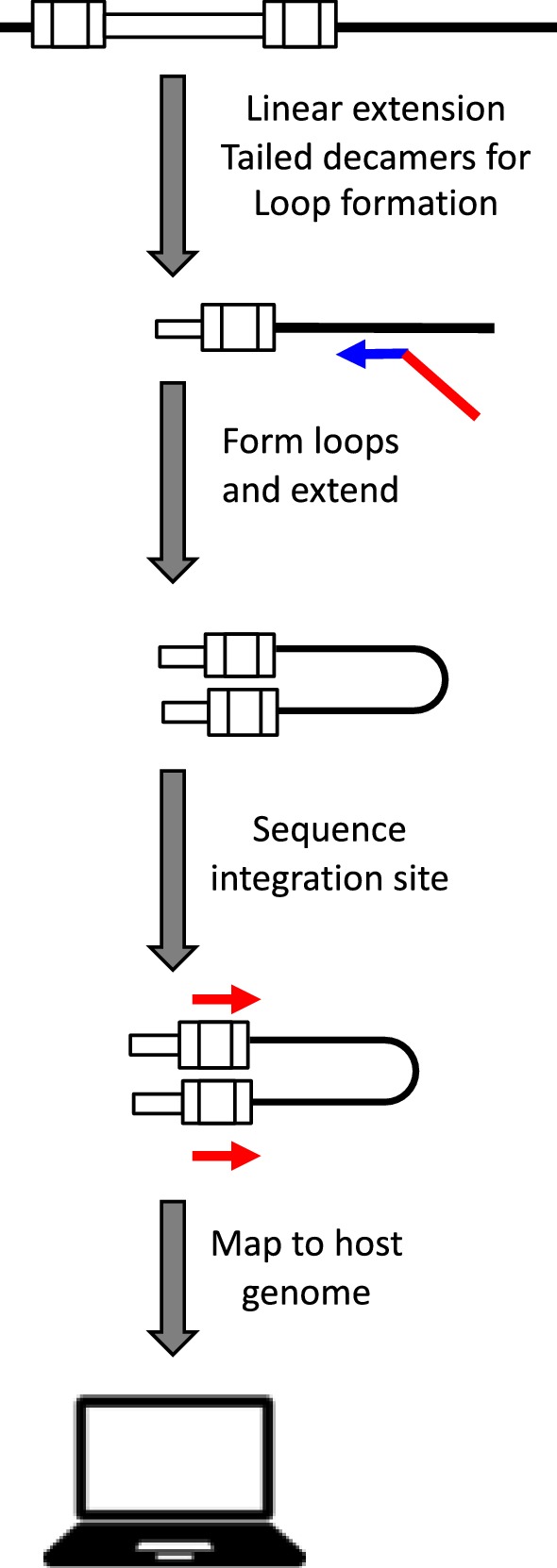
HIV integration site loop amplification (ISLA) assay workflow. HIV DNA copy numbers are quantified from extracted nucleic acid and diluted to an endpoint prior to linear extension using primers in HIV *env* and HIV *nef*, then random decamers (blue) tailed with an HIV LTR U5-specific sequence (red) are annealed to the linear template and extended, the single stranded DNA downstream of the random decamer primer is removed and the U5-specific region anneals to its complementary sequence in the HIV LTR forming a loop which is then amplified, the resulting loop contains U5 sequence that is flanked by the host genome, using primers complementary to U5 the integration site can be mapped. Integration sites identified more than once indicate clonal expansion

New methods have been developed to detect and quantify HIV integration sites. Assays that can detect both the site of integration and the presence of clonal expansion represent a pivotal advance. Pioneering work from the Bangham laboratory inferred selective forces that shape the landscape of human T cell leukemia virus 1 (HTLV-1) clones in vivo [184]. A high-throughput approach was developed to identify the locations of unique HTLV-1 integration sites in the host genome [185]. This method, based on random shearing and linker-mediated PCR followed by next generation paired-end sequencing, enables simultaneous mapping and quantification of unique integration sites in HTLV-1 infected T-cells [185, 186]. Integration sites from gene therapy vectors and retroviruses, including HTLV-2 [187], murine leukemia virus (MLV) [188], and recently HIV [78], have been investigated using this approach. The abundance of specific clones can be assessed by the number of unique host break points. Identical integration sites with different lengths of host sequence imply clonal expansion, whereas identical integration sites with identical lengths of host sequences are the product of PCR amplification (Fig. 3). A novel alternative approach to identify HIV proviral integration sites, the integration site loop amplification (ISLA) assay, was developed by Wagner and co-workers [80] (Fig. 4). ISLA utilizes linear amplification of proviral integration sites to increase their abundance, followed by loop formation using random decamers tailed with an HIV LTR U5-specific sequence [80]. This results in circularized amplicons containing HIV LTR sequence flanking the host genome at the site of integration, the HIV:host junction is then mapped using HIV LTR primers (Fig. 4). Both of these methods (reviewed in [41]) reduce bias since they do not rely on PCR amplification or restriction digestion both of which favor amplification of some integration sites.

Critically, these assays identify the integration junction sequence and the presence of clonal expansion. Yet, current approaches for integration site identification do not characterize the structure of the provirus located at the integration site. This is mainly due to the short amplicon constraints imposed by current next generation sequencing platforms. Integration site recovery has been insightful and has retrieved 10^2^–10^3^ integration sites from 5 to 10 million PBMCs. Initial studies revealed a number of unexpected findings: HIV infected cells present after prolonged cART are frequently clonally expanded. Overall, circa 40% of all cells harboring HIV proviruses are the product of clonal expansion. As described above, the efficiency of recovery of integrated proviruses is comparatively low, as such the actual frequency of clonal expansion is likely to be much higher [78]. Moreover, Wagner et al. demonstrated that clonal expansion increased during antiretroviral therapy [80]. Longitudinal analyses revealed specific expanded clones were present over prolonged periods (> 10 years), demonstrating durable persistence of HIV infected cells [80].

Analysis of the distribution of integration sites using bioinformatic tools to investigate the functions of genes have revealed many proviruses were present in genes associated with cell growth [78, 80]. The cells remaining after long-term cART were infected many years prior to their sampling. Therefore, the enrichment in genes associated with cell growth raises the strong possibility that the presence of the provirus in these genes contributes to persistence, expansion, or both.

As expected, proviruses were most frequently identified in introns, and were integrated in the same or opposite direction of host transcription, similar to those detected in in vitro infections. However, proviruses in several genes, including *BACH2* and *MKL2*, were present integrated only in the same orientation as the host gene transcription. In addition, integrations into these genes were highly restricted, and identified only in a limited region of the host gene (Introns 4 and 6 for *MKL2*, Introns 4 and 5 for *BACH2*) [78]. Control experiments analyzing the distribution of HIV integration sites in acute in vitro infections of HIV demonstrated that proviruses are commonly found throughout the *MKL2* and *BACH2* genes [78]. However, the striking finding that in in vivo experiments they were only present in the same orientation as host gene transcription after prolonged cART suggested that proviruses present in intron 4 or 6 provided a direct selective advantage that contributed to persistence, and expansion [78]. Proviruses present in other parts of these genes were not detected after prolonged ART presumably because they did not have a selective advantage.

Megakaryoblastic Leukemia (MKL)/Myocardin-Like Protein 2 (MKL2) is a phosphorylation mediated transcriptional activator that modulates the transcription of many cellular early genes by regulating the transcription factor serum response factor (SRF). SRF is a reported oncogene involved in promoting proliferation of mammary and hepatocellular adenocarcinomas [189, 190]. Fusions of *MKL2* and *C11orf95* have been frequently identified in choroid lipomas, suggesting a role in growth and expansion of these neoplasms [191]. *MKL2* fusion with *RREB1* has been described in oropharyngeal sarcoma [192]. MKL2 has also been implicated in development of hippocampal neurons [193] and muscle [194, 195]. However, the precise role of MKL2 in T cell homeostasis has not been extensively studied.

The transcription regulator protein BACH2 is a member of the basic leucine zipper transcription factor family that typically associates with Maf proteins to permit the binding of a BACH2-Maf heterodimer to specific DNA promoter recognition sites (reviewed by Igarashi et al. [196]). BACH2 functions in normal B cell development [197], is frequently deleted in B cell tumors [183, 198], and reduced levels of BACH2 have been associated with poor outcome in response to chemotherapy [199]. In addition, aggressive lymphomas containing IGHCδ-BACH2 fusion protein have been identified [200]. More recently, BACH2 has been demonstrated to have critical roles in T cell homeostasis [201–203]. As reviewed by Richer et al. [204], BACH2 may participate in regulating development during T cell differentiation, especially of T-regulatory and T effector lineages. BACH2 may also contribute to maintaining cell quiescence by preventing differentiation into effector memory cells [201, 202]. It is not known how HIV integration affects *BACH2* expression.

In infected cells, integrations into *BACH2* were limited to introns 4 and 5, which are in the 5′ untranslated region several thousand nucleotides upstream of the *BACH2* start codon [78]. This suggests the possibility that transcription may be initiated from the proviral LTR promoter and not from the authentic *BACH2* promoter. Indeed, Cesana et al. recently reported the detection of chimeric transcripts encoding HIV-LTR-*BACH2* in a substantial number of HIV infected patients undergoing antiretroviral therapy [205]. These transcripts consists of HIV 5′ untranslated sequence to the major splice donor from HIV spliced to exon 5 of *BACH2*. It is not yet clear whether these transcripts are initiated at +1 of HIV or represent read-through transcripts of *BACH2* intron 4 [205]. These data demonstrate that chimeric host–HIV RNA is common, and increased expression of *BACH2* may influence persistence and clonal expansion. Cesana et al. also demonstrated evidence of chimeric HIV transcripts with *STAT5B*, a transcription factor central to T cell activation, in PBMCs from a substantial number of infected individuals undergoing antiretroviral therapy [205]. Integrations into *STAT5B* were identified in a number of patients, but without significant orientation specificity [78]. Additional study of these specific examples of HIV integration is needed. Although integrations into these genes has been demonstrated, only limited sequence information of the HIV:host junction has been obtained and the structure of the entire proviruses in *BACH2*, *MKL2* and *STAT5B* remains uncertain. The data of Cesana et al. indicate that at least the R, U5, and 5′ untranslated HIV sequence to the major splice donor is present, but the remainder of the provirus structure is not known. It should be emphasized that although integrants in these genes were found in numerous HIV infected individuals, their actual abundance in PBMC populations is quite low, on the order of 1–10 copies/million PBMC, complicating amplification and characterization of integrated proviruses.

The limits and consequences of clonal expansion remain poorly understood. Clonal expansion is detected during long-term cART, although total HIV DNA levels remain relatively constant. Thus, clonal expansions occur but do not appear to increase the abundance of virus infected cells. Control mechanisms that permit clonal expansion but restrict the number of HIV infected cells are not known. Similarly, HIV integration has not been associated, as yet, with malignant transformation of HIV infected cells. In fact, CD4+ T cell leukemia and lymphoma is distinctly uncommon in HIV infected individuals [206, 207]. It is likely that clonal amplification, even to the large abundance as we and others have identified [78, 80, 208], is insufficient for malignant transformation. Of note, clonal expansions of T cells are present in individuals infected with other human retroviruses, including both HTLV-1 and HTLV-2 [185, 187]. However, hematologic malignancies are only detected in a minority of HTLV-1 infections, suggesting that malignant transformation is likely due to additional requirements [209].

Advances in next generation sequencing approaches have enabled in depth analyses of proviral integration sites from PBMCs of HIV infected individuals on cART [78, 80, 208]. These methodologies allow quantification of multiple identical integration sites and the ability to identify clonal expansion. Since the probability of HIV integration into the exact same location in the host genome more than once is vanishingly small, clonal expansion can be defined as a population of cells derived from cell division that harbor a provirus integrated into the exact same location in the host genome. Analyses of these integration sites show clonally expanded HIV infected CD4+ T cells exist after years on therapy suggesting that clonal expansion is a major mechanism that enables HIV persistence despite the success of cART [78]. Yet, current approaches for integration site identification do not characterize the structure of the provirus located at the integration site. This is mainly due to the short amplicon constraints imposed by these sequencing platforms. Therefore, novel approaches to map integration sites and provirus structure will aid in understanding long-term HIV persistence and reservoir maintenance. Characterization of HIV integrant structures will be useful for constructing model systems in which proviruses can be specifically targeted, for instance with CRISPR/Cas, to investigate the effects of proviral integration on cell growth and differentiation. Further detailed analyses of integration site distribution in vivo will aid in the study of cellular functions in the context of HIV infection. As integration sites are identified by various research groups, they should be compiled and made available for analyses through established public databases in order to robustly advance this key area of inquiry [210].

## The role of clonal expansion in maintaining HIV persistence

Clonal expansion of HIV infected cells can persist in patients for over 10 years on suppressive cART [78, 80, 208]. Early studies found populations of virus with identical sequences emerge in the plasma of HIV infected individuals who were suppressed for years on cART suggesting that highly expanded cell clones gave rise to persistent viremia [211, 212]. The discovery of identical HIV sequences from clearly defective or APOBEC hypermutated proviruses indicated that the only way the virus could arise was through clonal expansion [213]. A mathematical model predicted that clonal expansion and contraction of latently infected cells upon sporadic antigen stimulation can generate persistent low level viremia and lead to intermittent viral blips [214]. Although experimental data is needed to confirm these findings, this model also indicates that a fraction of activated T cells can revert back to the latent state thereby providing a mechanism to continually replenish the latent reservoir [214].

The majority of HIV DNA decay occurs within the first year on cART, after which it remains relatively stable in participants treated during chronic infection [28]. Meanwhile, the reservoir of replication competent proviruses, as measured with qVOA, decays minimally [26]. Yet, the frequency of clonally expanded cells harboring integrated proviruses increase over time [80]. Therefore, the overall composition of the reservoir is dynamic and changes over time despite suppressive cART. For instance, although the majority of integrated proviruses are defective or deleted [23], some can still be transcribed and produce proteins which can be targeted by CTL for killing [24, 25]. Moreover, clonal expansion of cells harboring integrated proviruses can occur through homeostatic forces, as a consequence of the integration site, or by antigen stimulation [78, 79].

Homeostatic proliferation is a mechanism for T cell division that may play a role in maintaining the reservoir over time. Previous studies have implicated interleukin 7 (IL-7) in the homeostatic regulation of the T cell pool [215]. IL-7 is produced by non-hematopoietic cells and is involved in thymocyte development and survival [216]. During chronic infection, CD4+ T cell depletion is associated with increased levels of proliferation through elevated levels of IL-7 and ultimately larger reservoir size, indicating that IL-7 is responsible for the persistence of latently infected cells by promoting homeostatic proliferation [217]. IL-7 induced proliferation can occur without reactivation of the virus in an in vitro model of HIV latency [89] and in vivo [88]. Taken together these studies suggest T cell division of HIV infected cells permits HIV persistence in the absence of ongoing cycles of viral replication.

Integration site driven clonal expansion is believed to occur infrequently and is the result of a nearly random integration site selection process. Multiple individuals have been identified as having proviral integrations enriched in genes associated with cell growth some of which were found to be clonally expanded [78, 80]. These findings raises the possibility that the presence of the provirus within the oncogene contributes to the ability of the cell to persist or to undergo clonal expansion in an integration site driven manner. The frequency of integration site driven clonal expansion and the mechanisms that govern these cell clones are still under active investigation.

Identifying clonal populations containing replication-competent HIV proviruses is challenging because these cells are generally rare, and are present in large populations of cells containing defective proviruses. In vivo, most HIV infected cells persisting for prolonged periods on ART contain defective proviruses [23, 111]. The initial finding that many cells present after prolonged cART are the products of clonal expansion [78, 80] was thought to reflect clonal expansion of defective, but not replication competent proviruses [208]. Clonal populations harboring defective HIV can contribute to ongoing immune activation, which may enable persistence [24, 25, 111] but these populations cannot give rise to rebounding viremia upon treatment interruption and therefore do not contribute to the ‘true’ HIV reservoir. Initial analyses of plasma HIV during prolonged antiretroviral therapy revealed the presence of populations of identical sequences, suggesting these variants were the product of clonal expansion. Detailed analyses of one example of predominant plasma clone [79] led to identification of the integration site of the provirus responsible for the clone and that the provirus was replication competent. The provirus has a unique integration site, but is present in a region that has not been mapped to a unique location. The integrant was designated AMBI-1 (ambiguous) to reflect that the location in the human genome is ambiguous [79]. Amplification from the known integrant was determined to be replication competent in in vitro infections, and the identical virus could also be repeatedly recovered in vitro from endpoint diluted PBMC cultures. Cells harboring the AMBI-1 integrant were found to be widely anatomically distributed but enriched in cancer metastases indicating that the clone expanded in response to the cancer antigen [79]. These data demonstrated that clonally expanded populations can contain infectious HIV, and therefore represent a relevant reservoir for HIV during cART.

The finding of a clonally expanded population with infectious HIV was unexpected as HIV is frequently cytolytic and encodes an accessory protein (Vpr) which can arrest the cell cycle [218]. It is possible that cell division and virus production are compartmentalized, and do not take place concurrently. Recent studies have shown that populations of clonally expanded cells persist on cART and only a fraction of cells within the clone are transcriptionally active [219, 229]. Furthermore, upon treatment interruption, transcriptionally active cells ultimately gave rise to rebounding viremia [220]. Taken together, these studies suggest that clonally expanded cells containing replication competent proviruses comprise a portion of the true HIV reservoir and that a proportion of transcriptionally active cells within the clone contribute to low level persistent viremia and ultimately rebounding virus upon treatment interruption. A critical understanding of these populations, their HIV RNA expression levels, and mechanisms which govern their active or latent states are is crucial for targeting eradication efforts.

The frequency of clonally expanded cells that harbor replication competent proviruses, such as AMBI-1, is not known, although recent data indicate that they may be relatively common [221–223]. Unequivocal identification of such proviruses is labor intensive and technically complex, but their characterization will yield key information regarding the requirements for persistence during therapy. Such proviruses represent a substantial obstacle to HIV cure. Furthermore, the dynamics of clonal expansion of cells containing replication competent proviruses is not well described and may be shaped by immune selection pressures. Recently it was found that these clones can wax and wane or persist steadily in vivo for years [224]. The mechanisms by which these cells can proliferate without viral reactivation to maintain the reservoir despite therapy poses a major obstacle towards an HIV cure. Shock and kill strategies aimed at HIV eradication will need to reactivate quiescent cells without inducing cell replication, which could result in unintended expansion of a cellular reservoir of infected cells. A number of such agents capable of activating cells without inducing cell division are under investigation. Analysis of proviral integration sites as part of the analytic approach to HIV eradication strategies will be a useful adjunct to current reservoir studies. Current integration site assays are, as described above, not highly efficient, and sensitivity will likely need to be optimized to detect low level clonal expansion. Taken together, these findings suggests that both active CTL selection pressures and passive clonal expansion mechanisms can drive the remodeling of the HIV reservoir over time. Finally, clonal expansion provides multiple targets to decrease the probability that a cell with an intact provirus will be eliminated precluding eradication strategies.

## Characterizing clonal expansion in the setting of eradication strategies

Several strategies aimed at eradicating the latent HIV reservoir have been employed. These include ART regime intensification, gene therapy, stem cell transplantation, therapeutic vaccines, and latency reversal agents (LRAs). LRAs are being used in a number of studies to potentially eliminate HIV through inducing reactivation of quiescent T cells in the hopes that these reactivated cells will undergo cell death. The original concept of purging the latent reservoir by reversing latency through activation of latently infected cells was implemented using interleukin 2 (IL-2) and T cell activators such as anti-CD3 antibodies (OKT3) [225, 226]. From these initial studies, it was clear that activation of latently infected T cells could be achieved and may enable purging of the reservoir, however, other compounds to reverse latency with reduced toxicity were needed.

Characterizing clonal expansion in the setting of eradication strategies such as ‘shock and kill’ sheds critical new light on the true structure of the HIV reservoir and whether that structure has been altered with treatment. The majority of current LRA strategies have utilized histone deacetylase inhibitors (HDACi). Even though some LRA strategies have successfully reversed latency in patients undergoing suppressed cART, measured by increased HIV transcription and virion production, no strategy has led to a decrease in the frequency of latently infected cells to date (reviewed by Bashiri et al. [227]). The inability of current LRA strategies to reduce the latent reservoir size can be attributed to insufficient host immune responses after latency reversal, an insufficient magnitude of latency reversal, or both. Therefore, new strategies that have higher specificity and potency to efficiently reverse latency may be needed in combination with therapies aimed at boosting the host immune response to sufficiently clear virus producing cells [228].

It is possible that LRA treatment can instead promote clonal expansion and thereby increase the reservoir size preventing elimination. IL-7 therapy has been administered to HIV-infected individuals to induce an increase in naïve and memory T-cell numbers [86, 87]. Yet, in vitro and in vivo studies predict that IL-7 administration would lead to an expansion of T-cells including HIV infected T-cells and thereby have a potential to increase the HIV reservoir without reactivating the virus [88, 89]. Characterization of individual HIV integration sites will identify which integrants were reactivated, eliminated, or expanded during latency reversal.

## Conclusions

Integration is a critical and, as yet, irreversible step in HIV replication that enables the persistence of HIV in a reservoir of long-lived cells despite suppressive antiretroviral therapy. The reservoir of infected cells harboring inducible full length replication competent proviruses is a major barrier to an HIV cure. Understanding the mechanisms of reservoir maintenance may provide novel targets for therapeutic interventions. Clonal expansion of HIV infected cells is a key mechanism for maintenance of the reservoir.

Current assays to measure and characterize integration sites are costly, time consuming, and labor intensive. Therefore, novel assays to measure clonal expansion are of key interest. Alternatively, sequences can be obtained from individual HIV proviruses through endpoint dilution and PCR amplification [229]. While it is impossible to determine whether two proviruses are identical without comparing individual full length sequences, which are prohibitively expensive to generate at present, a surrogate to predict clonal expansion can be calculated with the clonal prediction score [230]. This metric considers the length of the amplicon and the intra-patient genetic diversity to determine the likelihood that individual identical sequences are the result of clonal expansion. This tool, while not definitive, may provide a measure to assess clonal expansion in the absence of intensive integration site analyses.

Methods to characterize the provirus sequence and structure as it is integrated into particular locations in the host genome need further development. For example, the generation of full length HIV genome amplicons that cross into the host at the HIV-host junction could provide insights into the abundance of replication competent proviruses in clonal populations, as well as the biological relevance of enriched integration sites. Extensive sequence data will enable phylogenetic analyses to elucidate timing of proviral integration as well as estimates of total population sizes within the host. Detailed assessments of intact versus defective and deleted proviruses can characterize the composition of HIV reservoirs over time and linking these data to the integration site may reveal novel immune selective pressures that eliminate or favor certain proviral structures over time.

Distinguishing how proviral structure influences transcription and RNA splicing within individual host genes may reveal alternative splice variants and their biological function in HIV persistence. For instance, it has been shown that HIV and lentiviral vectors may induce aberrant RNA splicing mechanisms resulting in the production of chimeric transcripts containing HIV sequence fused to cellular exon sequences [231–233]. Furthermore, it has been shown that lentiviral vectors with active LTRs can induce neoplastic transformation through the activation of cancer-related genes via promoter insertion [234]. In addition, chimeric HIV/*BACH2* transcripts were found in several individuals (34%) with HIV integrations in the *BACH2* gene, indicating that expression of these transcripts could favor the persistence of those cells [205]. Likewise studying the three-dimensional (3D) chromatin structure of integrated proviruses may provide insights into mechanisms influencing the location of integration as well as the 3D interactions between integrated proviruses and host genes.

Finally, elucidating the timing of clonal expansion may provide novel strategies to limit the size of the reservoir in HIV infected individuals. For instance, the extent of clonal expansion prior to the initiation of treatment and the effects of early treatment on the pool of infected, clonally expanded cells is of great interest. Understanding whether antiretroviral treatment permits clonal expansion or rather reveals the infected cell clones that were present prior to and upon treatment initiation is pivotal. Such studies require the development of deeper and more comprehensive integration site mapping techniques and the examination of unique cohorts of individuals identified during acute HIV infection. Characterizing clonal expansion in the setting of immune recovery is needed to determine whether the increase in CD4 cell number over time during therapy is reflected in clonally expanded populations. Gaining a deeper understanding of clonal expansion of HIV infected cells as a mechanism of HIV persistence despite cART will provide needed strategies for reservoir elimination and ultimately HIV eradication.

## Abbreviations

cART: combination antiretroviral therapy
PBMC: peripheral blood mononuclear cell
GALT: gut-associated lymphoid tissue
CTL: cytotoxic T lymphocyte
APOBEC: apolipoprotein B mRNA editing enzyme
ddPCR: droplet digital PCR
qVOA: quantitative viral outgrowth assay
BrdU: bromodeoxyuridine
IN: HIV integrase
CA: HIV capsid
RT: reverse transcriptase
LTR: long terminal repeat
PIC: pre-integration complex
ALLINIs: allosteric integrase inhibitors
BAF1: barrier to autointegration factor 1
HMG: high mobility group
LAP2α: lamina-associated polypeptide 2α
TNPO3: karopherin transportin SR2
LEDGF/p75: lens epithelial-derived growth factor
TREC: T cell receptor excision DNA circles
ISLA: integration site loop amplification
ISA: integration site assay
AMBI-1: ambiguous integrant 1
*MKL2*: megakaryoblastic leukemia/myocardin-like protein 2
SRF: serum response factor
*STAT5B*: signal transducer and activator of transcription 5B
*BACH2*: BTB domain and CNC homolog 2
